# Sixfold improved single particle measurement of the magnetic moment of the antiproton

**DOI:** 10.1038/ncomms14084

**Published:** 2017-01-18

**Authors:** H. Nagahama, C. Smorra, S. Sellner, J. Harrington, T. Higuchi, M. J. Borchert, T. Tanaka, M. Besirli, A. Mooser, G. Schneider, K. Blaum, Y. Matsuda, C. Ospelkaus, W. Quint, J. Walz, Y. Yamazaki, S. Ulmer

**Affiliations:** 1 RIKEN, Ulmer Initiative Research Unit, 2-1 Hirosawa, Wako, Saitama 351-0198, Japan; 2 Graduate School of Arts and Sciences, University of Tokyo, Tokyo 153-8902, Japan; 3 CERN, CH-1211 Geneva 23, Switzerland; 4 Max-Planck-Institut für Kernphysik, Saupfercheckweg 1, 69117 Heidelberg, Germany; 5 Institut für Quantenoptik, Leibniz Universität Hannover, Welfengarten 1, 30167 Hannover, Germany; 6 Institut für Physik, Johannes Gutenberg-Universität, 55099 Mainz, Germany; 7 Physikalisch-Technische Bundesanstalt, QUEST, Bundesallee 100, 38116 Braunschweig, Germany; 8 GSI-Helmholtzzentrum für Schwerionenforschung GmbH, 64291 Darmstadt, Germany; 9 Helmholtz-Institut Mainz, sektion MAM, 55099 Mainz, Germany; 10 RIKEN, Atomic Physics Research Unit, 2-1 Hirosawa, Wako, Saitama 351-0198, Japan

## Abstract

Our current understanding of the Universe comes, among others, from particle physics and cosmology. In particle physics an almost perfect symmetry between matter and antimatter exists. On cosmological scales, however, a striking matter/antimatter imbalance is observed. This contradiction inspires comparisons of the fundamental properties of particles and antiparticles with high precision. Here we report on a measurement of the *g*-factor of the antiproton with a fractional precision of 0.8 parts per million at 95% confidence level. Our value 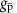/2=2.7928465(23) outperforms the previous best measurement by a factor of 6. The result is consistent with our proton *g*-factor measurement *g*_p_/2=2.792847350(9), and therefore agrees with the fundamental charge, parity, time (CPT) invariance of the Standard Model of particle physics. Additionally, our result improves coefficients of the standard model extension which discusses the sensitivity of experiments with respect to CPT violation by up to a factor of 20.

## Abstract (web summary)

High-precision measurements could disclose fundamental dissimilarities between matter and antimatter, which are found imbalanced in the Universe. Here, the authors measure the magnetic moment of the antiproton with six-fold higher accuracy than before, finding it consistent with that of the proton.

Precise tests of charge, parity, time (CPT) invariance^1^ are inspired by the intriguing lack of antimatter in our Universe^2^. Despite its importance to our understanding of nature, only a few direct precise tests of CPT symmetry are available^3^,^4^,^5^,^6^,^7^,^8^,^9^. Our experiments contribute such tests by comparing the fundamental properties of protons and antiprotons with high precision. Recently we performed the most precise comparison of the antiproton-to-proton charge-to-mass ratio 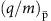/(*q*/*m*)_p_ with a fractional precision of 69 parts per trillion^10^. Here, we report an improved measurement of the magnetic moment of the antiproton 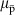.

Our comparisons are based on frequency measurements of single particles in cryogenic Penning traps. These traps employ the superposition of a strong magnetic field *B*_0_ in the axial direction and an electrostatic quadrupole potential^11^. Under such conditions, a trapped charged particle follows a stable trajectory consisting of three independent harmonic oscillator motions–the modified cyclotron frequency *ν*_+_, the magnetron frequency *ν*_−_, and the axial frequency *ν*_*z*_. Non-destructive measurements of these frequencies^12^ enable the determination of the cyclotron frequency 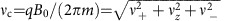 (ref. 13). Together with a determination of the spin-precession, or Larmor frequency *ν*_L_, the magnetic moment 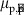 can be determined in units of the nuclear magneton *μ*_N_, where 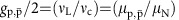 is the *g*-factor of the particle. The determination of the Larmor frequency relies on the detection of resonantly driven spin transitions by means of the continuous Stern-Gerlach effect^14^. An axial magnetic field *B*_*z*_=*B*_2_(*z*^2^−*ρ*^2^/2) is superimposed on the homogeneous magnetic field *B*_0_ of the trap. This magnetic bottle couples the spin magnetic moment to the axial oscillation frequency *ν*_*z*_ of the particle, which is directly accessible by measurement. When a spin-flip takes place, *ν*_*z*_ is shifted by 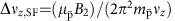. This technique has been applied in electron/positron magnetic moment measurements 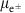
(ref. 3), however, its application to measure the magnetic moments of the proton/antiproton^15^,^16^ is much more challenging, since 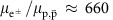. Therefore, to resolve single antiproton spin transitions an ultra-strong magnetic bottle is needed, we use *B*_2_=2.88·10^5^ T m^−2^.

In this article we report a direct measurement of the *g*-factor of a single antiproton, with a fractional precision of 0.8 p.p.m. Our result is based on six individual *g*-factor measurements and is 6 times more precise than the current best value^15^.

## Results

### Experimental set-up

**Figure 1:**
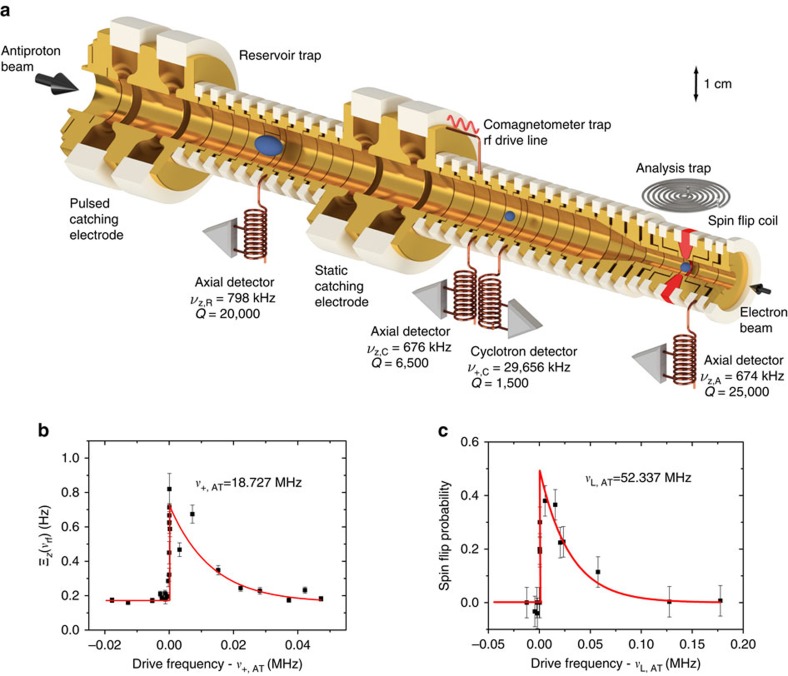
Experimental set-up and resonances. (**a**) Schematic of the Penning trap set-up used in the BASE experiment. A cloud of antiprotons is stored in the RT, which supplies single particles to the CT and the AT when required. The CT is used for continuous magnetic field measurements. The AT is the trap with the strong superimposed magnetic bottle, which is used to measure the cyclotron frequency and the Larmor frequency. All traps are equipped with radio-frequency excitation electronics and highly sensitive superconducting detection systems. (**b**) Cyclotron and (**c**) Larmor resonance curves, both measured in the AT. The error bars in (**b**,**c**) represent the standard deviations of the individual measurements.

The experiment^16^ is located at the antiproton decelerator (AD)^17^ facility of CERN. We operate a cryogenic multi-Penning trap system, see Fig. 1a, which is mounted in the horizontal bore of a superconducting magnet with field strength *B*_0_=1.945 T. Each Penning trap consists of a set of five cylindrical gold-plated electrodes in a carefully chosen geometry^18^. The individual traps are interconnected by transport electrodes. Application of voltage ramps to these electrodes enables adiabatic shuttling of the particles between the traps. Resonant superconducting tuned circuits with high quality factors *Q* (ref. 19) and effective temperatures of ≈8 K connected to specific electrodes enable resistive particle-cooling, non-destructive detection^12^ and measurements of the oscillation frequencies of the trapped antiprotons.

The entire trap assembly is mounted in a pinched-off vacuum chamber with a volume of 1.2l. A stainless steel degrader window, with a thickness of 25 μm, is placed on the vacuum flange, which closes the up-stream side of the chamber, being vacuum tight but partly transparent for the 5.3 MeV antiprotons provided by the AD. The chamber is cooled to cryogenic temperatures (6 K), where cryo-pumping produces a vacuum good enough to reach antiproton storage times of more than 1.2 years^20^.

We are using three traps, a reservoir trap (RT), a co-magnetometer trap (CT) and an analysis trap (AT). The RT with an inner diameter of 9 mm contains a cloud of antiprotons, which has been injected from the AD and supplies single particles to the other traps when needed^20^. This unique trap enables us to conduct antiproton experiments even during AD machine-shutdowns. The CT has the same geometry as the RT and is located 50 mm away from the AT. Single particle cyclotron frequency measurements at *ν*_+,CT_≈29.6 MHz allow for continuous sampling of the trap's magnetic field, with an absolute resolution of a few nanotesla. The AT has the strong superimposed magnetic bottle *B*_2_. The inner diameter is 3.6 mm, and the central ring electrode is made out of ferromagnetic Co/Fe material. This distorts the magnetic field in the centre of the trap such that *ν*_+,AT_≈18.727 MHz and *ν*_L,AT_≈52.337 MHz.

### *g*-factor measurement in the strong magnetic bottle

The strong *B*_2_ couples the spin magnetic moment 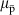 as well as the angular magnetic moment of the radial modes to its axial frequency *ν*_*z*,AT_ (*n*_+_, *n*_−_, *m*_s_)=*ν*_*z*,0_+Δ*ν*_*z*_(*n*_+_, *n*_−_, *m*_s_)≈674 kHz, where


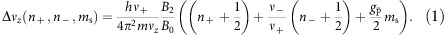


Here, *h* is Planck's constant, and *n*_+_ and *n*_−_ are the principal quantum numbers of the two radial modes, while 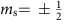 characterizes the eigenstate of the spin of the antiproton. A cyclotron quantum jump Δ*n*_+_=±1 changes the axial frequency by Δ*ν*_*z*,+_=±65 mHz, a transition Δ*n*_−_=±1 in the magnetron mode leads to Δ*ν*_*z*,−_=±42 μHz. A single spin transition, however, changes *ν*_*z*,AT_ by 183 mHz, which can be clearly detected if the changes in the quantum numbers of the radial modes are low enough to achieve a frequency stability of Δ*ν*_*z*_/*ν*_*z*,AT_≈10^−7^. This is considerably difficult since spurious voltage-noise *e*_n_ on the electrodes with a power spectrum density of 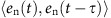 causes heating rates^21^;


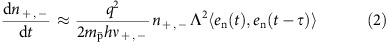


in the radial modes, where 1/Λ is a trap specific length. This parasitic heating leads to random walks in the radial modes and continuously changes the axial frequency *ν*_*z*,AT_. Voltage-noise densities of order *e*_n_=50 pV/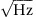 to 200 pV/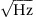 on the electrodes reproduce the observed axial frequency drifts. Note that d*n*_+,−_/d*t*∝*n*_+,−_. Therefore, the preparation of a particle with a sufficient axial frequency stability, which allows efficient detection of spin transitions, needs cooling of the cyclotron mode to sub-thermal energies, *E*_+_/*k*_B_<1.1 K (ref. 22).

The determination of the *g*-factor requires precise measurements of *ν*_+,AT_ and *ν*_L,AT_. To resolve these frequencies we apply radio-frequency drives to the trap and measure the axial frequency *ν*_*z*,AT_ of the trapped antiproton as a function of time and for different drive frequencies *ν*_rf_. Here the axial frequencies are determined as described in ref. 23. Once quantum transitions are resonantly driven, the axial frequency fluctuation Ξ_*z*_, defined as the s.d. of the difference of subsequent axial frequency measurements *σ*(*ν*_*z*,*k*+1_−*ν*_*z*,*k*_):=Ξ_*z*_, increases drastically, as shown in Fig. 1b,c (ref. 23). The shapes of these resonance lines


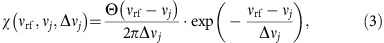


reflect the Boltzmann distribution of the axial energy due to the continuous interaction of the particle with the detection system. Here *ν*_*j*_ are the modified cyclotron frequency *ν*_+,AT_(*E*_*z*_=0)=*ν*_+,cut_ and the Larmor frequency *ν*_L,AT_(*E*_*z*_=0)=*ν*_L,cut_ at vanishing axial energy *E*_*z*_, respectively, Δ*ν*_*j*_∝*ν*_*j*_*·B*_2_·*T*_*z*_ is the line-width parameter^24^ and Θ(*ν*_rf_−*ν*_*j*_) the Heaviside function. The resolution which will eventually be achieved in the determination of the *g*-factor is consequently limited by the ability to resolve these two frequencies, *ν*_+,cut_ and *ν*_L,cut_.

To extract these frequencies, we scan the resonance lines only in a close range around the cut-frequencies. A random walk *ξ*(*t*), predominantly in the magnetron mode, continuously changes the magnetron radius *ρ*_−_(*t*), and as a result, the magnetic field experienced by the particle. This softens the slope of the resonance lines close to *ν*_+,cut_ and *ν*_L,cut_.

### Measurement procedure

To prepare the initial conditions of a *g*-factor measurement, we extract an antiproton from the reservoir and cool its modified cyclotron mode by resistive cooling in the CT. Subsequently we shuttle the particle to the AT. Using sideband coupling^25^ we first cool the energy of the magnetron mode to *E*_−_/*k*_B_<4 mK, then we determine the cyclotron energy by an axial frequency measurement, see equation (1). This sequence is repeated until *E*_+_/*k*_B_<1.1 K. For particles at such low cyclotron energies and axial frequency averaging times >90 s we achieve axial frequency fluctuations Ξ_*z*,back_<0.120 Hz. Next, we tune the particle to the centre of the magnetic bottle by adjusting offset voltages on the trap electrodes. This is crucial to suppress systematic shifts in the frequency measurements.

**Figure 2:**
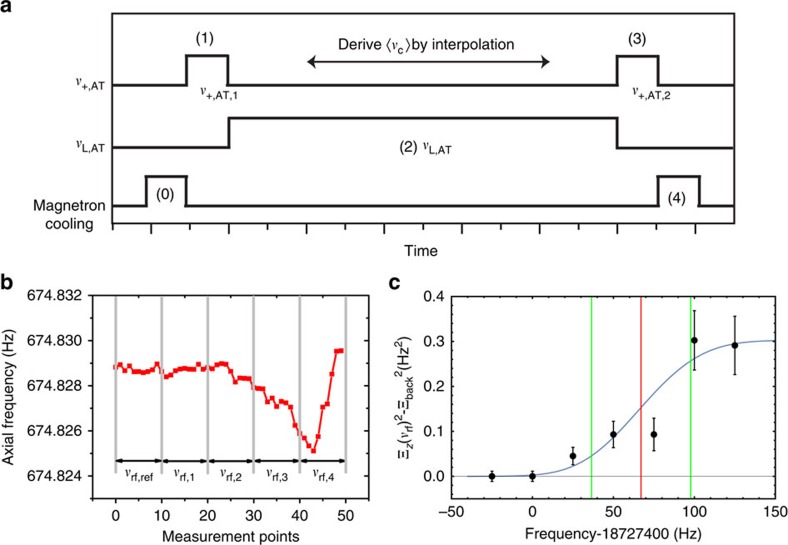
Experiment sequence and measurement of the cyclotron frequency. (**a**) We first centre the particle in the trap by cooling the magnetron motion to *E*_−_/*k*_B_<4 mK. Then we measure the modified cyclotron frequency *ν*_+,AT,1_. Subsequently we scan the Larmor resonance *ν*_L,AT_ and then measure the modified cyclotron frequency again *ν*_+,AT,2_. Afterwards we re-cool the magnetron motion. For a more detailed explanation of the experiment sequence we refer to the text. (**b**) Sequence of axial frequency measurements of 30 s averaging time while a radial dipolar drive at *ν*_rf,*k*_ is applied. The drive frequency is adjusted after each 10 measurements. Once the drive excites cyclotron transitions *ν*_rf,*k*_≈*ν*_+,cut_, the axial frequency fluctuation increases. (**c**) Projection of axial frequency data to axial frequency fluctuation Ξ_*z*_(*ν*_rf_). The error bars represent the standard deviations of the individual measurements. The red and green vertical lines indicate the determined mean value *ν*_+,cut_ and its 95% confidence level uncertainties, respectively, the blue solid line is a fit based on the the data-analysis described in the text.

Afterwards, we conduct the actual *g*-factor measurement as illustrated in Fig. 2a. This starts with (0) cooling of the magnetron motion, followed by (1) a measurement of the modified cyclotron frequency *ν*_+,AT,1_. Then (2) we scan the Larmor resonance, which typically takes 9–14 h. This is followed (3) by a second measurement of the modified cyclotron frequency *ν*_+,AT,2_. The cycle ends by (4) cooling of the magnetron motion.

To determine the modified cyclotron frequency, we apply a drive which induces on resonance a heating rate of d*n*_+_/d*t*(*ν*_rf_=*ν*_+,AT_(*E*_*z*_=0))≈4 s^−1^. We start with a background measurement at *ν*_rf,0_≈*ν*_+,AT_−100 Hz and then scan the drive frequency *ν*_rf_, typically in steps of 25 Hz over the resonance. For each individual drive frequency *ν*_rf,*k*_ we record ten axial frequency data-points, each averaged by *t*=30 s, and evaluate the axial frequency fluctuation Ξ_*z*_(*ν*_rf,*k*_). We repeat this scheme until the resonance line is clearly resolved, which means that for a resonant excitation frequency *ν*_rf,e_ the condition (Ξ_*z*_(*ν*_rf,e_)−Ξ_*z*,back_)/*σ*(ΔΞ_*z*_(*ν*_rf,e_), ΔΞ_*z*,back_)>3 is fulfilled. Here ΔΞ_*z*_(*ν*_rf,*k*_)=Ξ_*z*_(*ν*_rf,*k*_)/(2*N*−2)^0.5^ is the 68% confidence interval of the measurement, *N* is the number of accumulated data points per drive frequency *ν*_rf,*k*_ and *σ*(ΔΞ_*z*_(*ν*_rf,e_), ΔΞ_*z*,back_) the propagated s.e. of Ξ_*z*_(*ν*_rf,e_)−Ξ_*z*,back_. As an example a sequence of 50 axial frequency measurements with applied rf drives at *ν*_rf,*k*_ is shown in Fig. 2b. The first two data sets at *ν*_rf,1_ and *ν*_rf,2_ are consistent with the un-driven background fluctuation. At *ν*_rf,3_ and *ν*_rf,4_ the applied rf-drive induces cyclotron quantum transitions which clearly increases the measured axial frequency fluctuation to 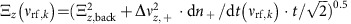. Figure 2c displays a projection of measured axial frequencies of an entire measurement sequence to axial frequency fluctuation Ξ_*z*_(*ν*_rf,*k*_) as a function of the applied rf-drive frequency.

The measured distribution of points Ξ_*z*_(*ν*_rf,*k*_) constrains the random walk *ξ*_−_(*t*) in the magnetron mode which has taken place during the frequency scan. Each individual measurement can be associated to a gaussian sub-distribution *w*_*k*_(*ν*, *ν*_+_(0)+*ξ*_−_(*t*))


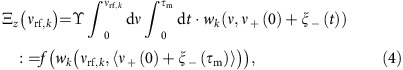


where 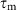 is the measurement time, ɛ a scaling factor, and *ν*_+_(0)+*ξ*_−_(*t*) the time dependent modified cyclotron frequency while *ν*_rf,*k*_ was irradiated. We reconstruct the distribution of modified cyclotron frequencies during the entire measurement 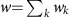 by minimizing 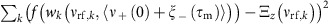 with the strength of the walk *ξ*_−_ as a free parameter. From the reconstructed distribution we evaluate *ν*_+,AT,1_ and derive the 95% confidence interval based on *w*. We back-up this treatment by Monte-Carlo simulations which model the exact measurement sequence. From the measurement shown in Fig. 2c, we extract *ν*_+,AT,1_=18,727,467(33) Hz, the mean value is indicated by the red vertical line, the green lines represent the 95% confidence interval of the measured mean, the blue solid line is the unperturbed line convoluted with the reconstructed *w*-distribution. The cyclotron frequency *ν*_c,AT,1_ is obtained by approximating 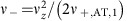, and using the invariance theorem^13^.

The Larmor frequency *ν*_L,AT_ is measured as first reported in ref. 23. First we average the axial detector transients for 90 s and determine the axial frequency *ν*_*z*,1_. Subsequently an off-resonant radio-frequency drive at frequency *ν*_rf,0_<*ν*_L_(*E*_*z*_=0) is irradiated and the axial frequency is measured again *ν*_*z*,2_. This scheme is repeated twice, with the rf-drive being tuned to values *ν*_rf,1_ and *ν*_rf,2_. Both frequencies are chosen to be close to the spin-resonance *ν*_rf,1_≈*ν*_rf,2_≈*ν*_L_(*E*_*z*_=0). Repetition of this measurement sequence for *N* times enables us to determine the fluctuations Ξ_*z*,back_, Ξ_*z*_(*ν*_rf,1_) and Ξ_*z*_(*ν*_rf,2_) the statistical significance being defined as in the cyclotron measurements. Once spin flips are driven by *ν*_rf,*k*_ the 183 mHz axial frequency jumps induced by the spin transitions add up to the background frequency fluctuation Ξ_*z*,back_. In this case the axial frequency fluctuation results in 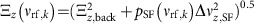, where *p*_SF_(*ν*_rf,*k*_) is the spin-flip probability^24^.

**Figure 3:**
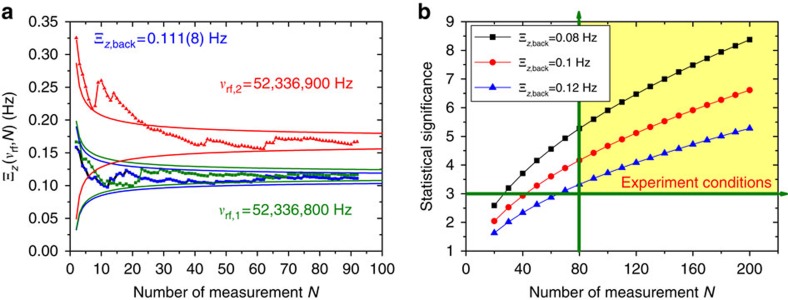
Measurement of the Larmor frequency. (**a**) Cumulative measured axial frequency fluctuation Ξ_*z*_(*ν*_rf_) for a background measurement and two different spin-flip drive frequencies at *ν*_rf,1_=52,336,800 Hz and *ν*_rf,2_=52,336,900 Hz. The blue data points reflect the background measurement, the green and the red points display Ξ_*z*_(*ν*_rf,1_) and Ξ_*z*_(*ν*_rf,2_), respectively. The solid lines indicate the 68% confidence intervals of the measurements. (**b**) Statistical significance (Ξ_*z*_(*ν*_rf_)−Ξ_*z*,back_)/(*σ*(ΔΞ_*z*_(*ν*_rf_), ΔΞ_*z*,back_) for three different background fluctuations Ξ_*z*,back_ and as a function of the number of accumulated measurements. For all frequency determinations which contribute to the *g*-factor evaluation the experiment was operated under the conditions highlighted by the yellow background, where (Ξ_*z*_(*ν*_rf_)−Ξ_*z*,back_)>3*σ*(ΔΞ_*z*_(*ν*_rf_), ΔΞ_*z*,back_).

Figure 3a shows results of such a measurement. The blue data-points represent the cumulative axial frequency background fluctuation Ξ_*z*,back_. The green data points display Ξ_*z*_(*N*, *ν*_rf,1_), measured while an rf-drive at *ν*_rf,1_=52,336,800 Hz was applied. The red data points show Ξ_*z*_(*N*, *ν*_rf,2_) where the rf-drive was tuned to *ν*_rf,2_=52,336,900 Hz. The solid lines represent the 68% confidence intervals of Ξ_*z*_(*N*, *ν*_rf,*k*_). From the measurement shown in Fig. 3a we extract after accumulation of *N*=93 data points Ξ_*z*,back_=0.111(8) Hz, Ξ_*z*_(*ν*_rf,1_)=0.116(8) Hz and Ξ_*z*_(*ν*_rf,2_)=0.168(13)Hz, which corresponds to a detection of spin transitions with >3.5*σ* statistical significance. Figure 3b shows the statistical significance (Ξ_*z*_(*ν*_rf_)−Ξ_*z*,back_)/(*σ*(ΔΞ_*z*_(*ν*_rf_), ΔΞ_*z*,back_)) for three different background fluctuations Ξ_*z*,back_ and as a function of the number of accumulated measurements. With the conditions of the experiment Ξ_*z*,back_<0.120 Hz and spin transitions which are driven at 50% probability we achieve a statistical significance >3.5*σ* by accumulating at least 80 measurements. In the experiment this is the minimum of data points which has been accumulated per irradiated *ν*_rf_.

Based on the above measurement we extract the Larmor frequency *ν*_L,AT_ as the arithmetic mean of *ν*_rf,1_ and *ν*_rf,2_. To define the 95% confidence interval we run Monte-Carlo simulations with defined parameters 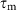 and *ν*_+,1_−*ν*_+,2_. The start frequency *ν*_+,1_ and the strength of the magnetron walk *ξ*_−_(*t*) are varied. We accept random walks which reproduce our result that within the 68% confidence bands Ξ_*z*_(*ν*_rf,1_)=Ξ_*z*,back_, and (Ξ_*z*_(*ν*_rf,2_)−Ξ_*z*,back_)/*σ*(Ξ_*z*_(*ν*_rf,2_), Ξ_*z*,back_)>3.5. We calculate the mean frequency of the simulated walk <*ν*_L_> and compare to the arithmetic mean frequency *ν*_L,exp_ which would have been extracted from the measurement. By integrating the resulting distribution *w*(*ν*_L,exp_−<*ν*_+_>) we determine the 95% confidence level of *ν*_L,exp_. For further details we refer to the Supplementary Discussion. From the measurement shown in Fig. 3a we extract *ν*_L,AT_=52,336,850(33)Hz.

Using the measured frequencies *ν*_c,1_, *ν*_L,AT_ and *ν*_c,2_ the *g*-factor is evaluated by calculating *g*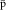/2=*ν*_L,AT_/<*ν*_c_>, where <*ν*_c_>=0.5·(*ν*_c,1_+*ν*_c,2_). This accounts for linear drifts in the magnetic field experienced by the particle during the scan of the Larmor frequency.

### Final result

**Figure 4:**
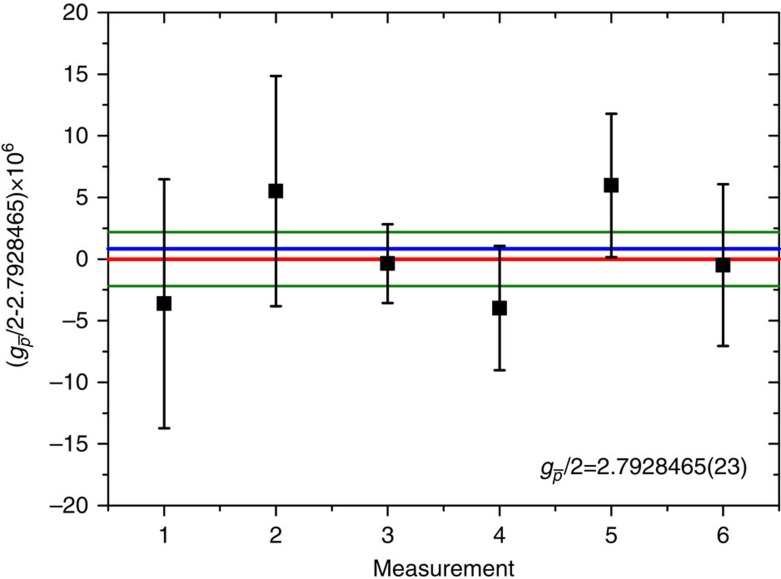
Experimental result. Results of the six *g*-factor measurements carried out during CERN's 2015/2016 accelerator shutdown between the 20 February 2016 and 5 March 2016. Based on this set of measurements we extract (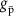/2)=2.7928465(23), as indicated by the red horizonal line. The green lines show the 95% confidence level error, the blue line represents the currently accepted value of the proton *g*-factor *g*_p_/2=2.792847350(9) (ref. 26). The error bars of the individual measurements are based on the uncertainties of the individual frequency measurements, which are dominated by the random walk in the magnetron mode.

We performed in total six *g*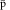-measurements, all of them were carried out during weekend- or night-shifts when magnetic field noise in the accelerator hall is small. The results are shown in Fig. 4. The uncertainties of the measurements are defined by the resolution achieved in the individual frequency measurements convolving the effects of magnetic field drift due to the magnetron random walk. To evaluate the final value of the *g*-factor we calculate the weighted mean of the entire data-set and extract


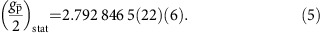


The first number in brackets represents the 95% confidence interval of the measured mean, the second number in brackets represents the scatter of the error according to *t*-test statistics.

Systematic errors come from non-linear drifts of the field of the superconducting magnet, drifts of the voltage source which is used to define the trapping potential and the random walk *ξ*_+_(*t*) in the modified cyclotron mode. From measurements with the co-magnetometer particle we estimate Δ*g*/*g*|_*B*0_≈0.015 p.p.m. Continuous voltage measurements constrain Δ*g*/*g*|_*V*_<0.001 p.p.m., while we obtain for the random walk in the cyclotron mode Δ*g*/*g*|_+_≈0.020 p.p.m. The non-linear contribution of the magnetron walk *ξ*_−_(*t*) to a systematic shift of the *g*-factor is implicitly considered in the primary data-evaluation of the measured resonance lines. We add these errors by standard error propagation and obtain


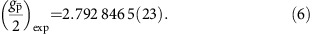


## Discussion

This result is consistent with our recently measured value of the magnetic moment of the proton in units of the nuclear magneton *g*_p_/2=2.792847350(9)^26^ and supports CPT invariance.

Our measurement also sets improved limits on parameters of the standard model extension (SME)^9^,^27^, which characterizes the sensitivity of a proton/antiproton *g*-factor comparison with respect to CPT violation. In a very recent comprehensive paper by Ding and Kostelecký^28^ the SME formalism is applied to Penning traps. There a detailed discussion on comparisons of experiments at different orientation and location is described. By adapting this work to our, data we derive for the leading coefficients described in the SME standard frame 
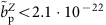
 GeV and 
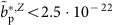
 GeV, which corresponds to a 11 and 22-fold improvement compared with the previously published constraints^28^. A summary of all upper limits on the SME-coefficients which are derived from our experiment are displayed in Table 1.

In our evaluation we have assumed that diurnal variations caused by the Earth's rotation average out. We neglect a potential bias which might be introduced by the fact that, due to maintenance of the apparatus, only a small amount of data was accumulated between noon and early afternoon. More equally distributed data accumulation will be addressed in our planned future experiments. For further details we refer to the Supplementary Discussion.

In summary, we have measured the magnetic moment of a single trapped antiproton in a single Penning trap with a superimposed magnetic bottle. The achieved fractional precision is at 0.8 p.p.m. (95% confidence level) and outperforms the fractional precision quoted in previous measurements by a factor of 6 (ref. 15). The precision of the result is limited by a background-noise driven random walk in the magnetron mode, which causes line-broadening. The measured antiproton *g*-factor is in agreement with our recent 3.3 p.p.b. proton *g*-factor measurement and supports CPT invariance. The much more precise proton measurements are based on the application of the challenging double Penning trap technique^26^. Implementation of this method to further improve the precision in antiproton magnetic moment measurements to the p.p.b. level will be targeted in our future research.

### Data availability

The data sets for the current study are available from the corresponding authors on reasonable request.

## Additional information

**How to cite this article:** Nagahama, H. *et al*. Sixfold improved single particle measurement of the magnetic moment of the antiproton. *Nat. Commun.* **8,** 14084 doi: 10.1038/ncomms14084 (2017).

**Publisher's note**: Springer Nature remains neutral with regard to jurisdictional claims in published maps and institutional affiliations.

## Supplementary Material

Supplementary Information Supplementary figures, supplementary discussion and supplementary references.

## Figures and Tables

**Table 1:** List of all SME-coefficients constrained by this measurement.

**Coefficient**	**Constraint**
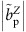	<2.1 × 10^−22^ GeV
	
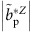	<2.6 × 10^−22^ GeV
	
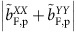	<1.2 × 10^−6^ GeV^−1^
	
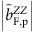	<8.8 × 10^−7^ GeV^−1^
	
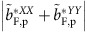	<8.3 × 10^−7^ GeV^−1^
	
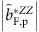	<3.0 × 10^−6^ GeV^−1^
