# Supplementary material for: Sixfold improved single particle measurement of the magnetic moment of the antiproton
Source: Nat Commun. 2017 Jan 18;8:14084. doi: 10.1038/ncomms14084 (PMC5253646; doi:10.1038/ncomms14084)
Supplement: Supplementary Information — Supplementary figures, supplementary discussion and supplementary references. [file ncomms14084-s1.pdf]

Supplementary Figures

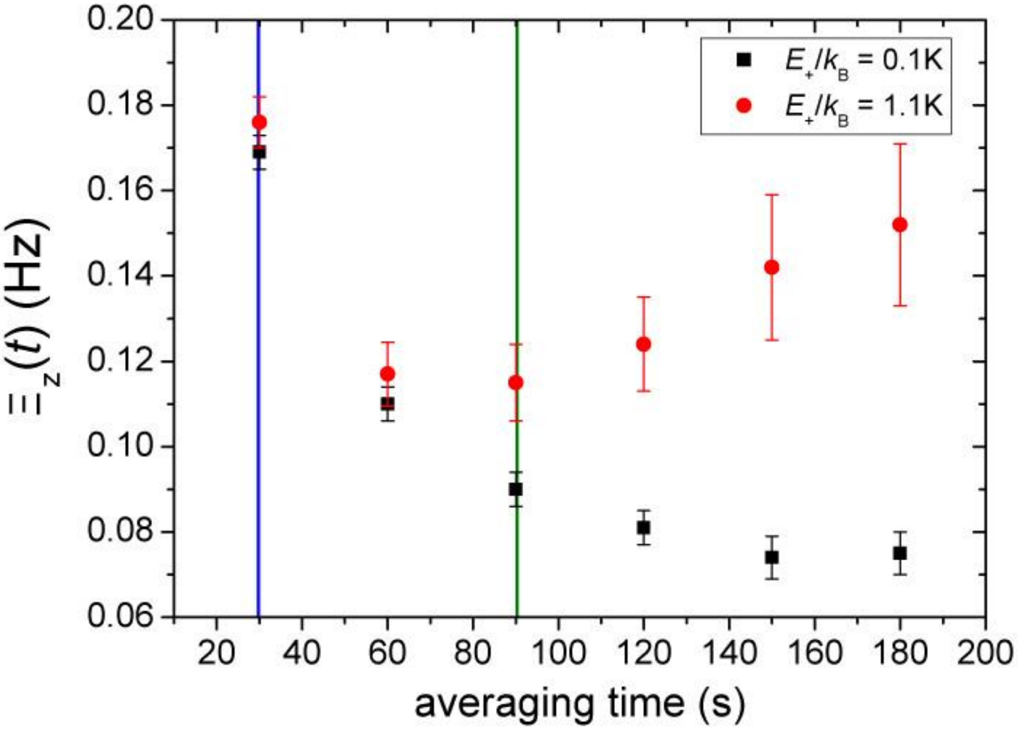

Supplementary Figure 1. Axial frequency fluctuation as a function of the averaging time. The black and the red data points represent axial frequency fluctuations $\Xi_{z,\mathrm{back}}$ measured with particles at cyclotron energies E_+/k_B of 0.1 K and 1.1 K, respectively. In measurements to resolve the modified cyclotron frequency we averaged the axial frequency for 30 s (blue line), in measurements to resolve the Larmor frequency we averaged the axial frequency for 90 s (green line). The error bars represent the statistical uncertainties of the individual measurements.

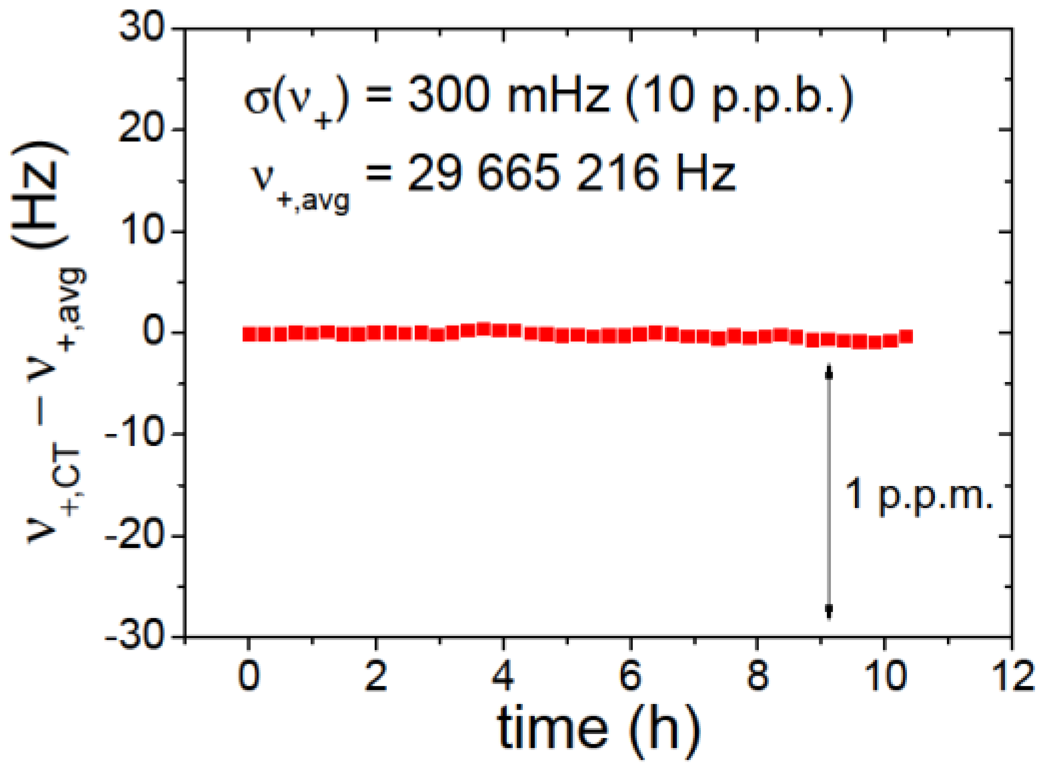

Supplementary Figure 2. Magnetic field measurement by using the co-magnetometer particle. The data set was measured while a Larmor frequency measurement was carried out in the analysis trap. Within the p.p.m. resolution reported in this paper, the magnetic field can be considered constant.

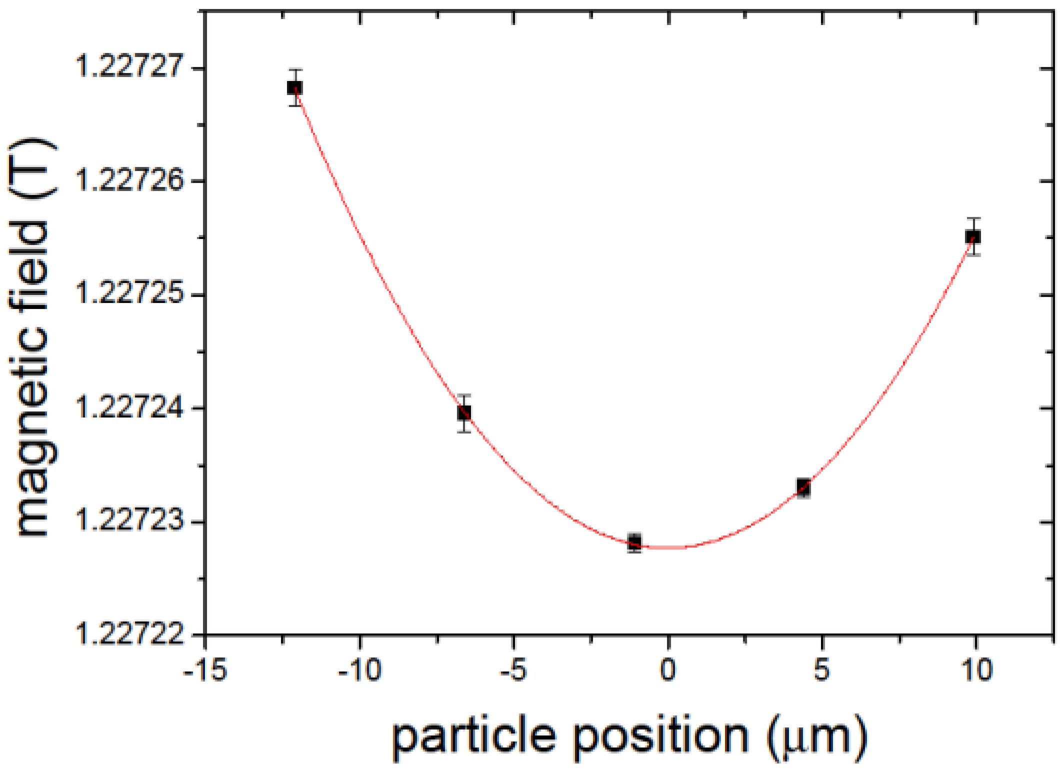

Supplementary Figure 3. Magnetic field as a function of position of the particle. The data are obtained by measuring the modified cyclotron frequency as a function of an offset voltage applied to one of the correction electrodes. The red line is a polynomial fit.

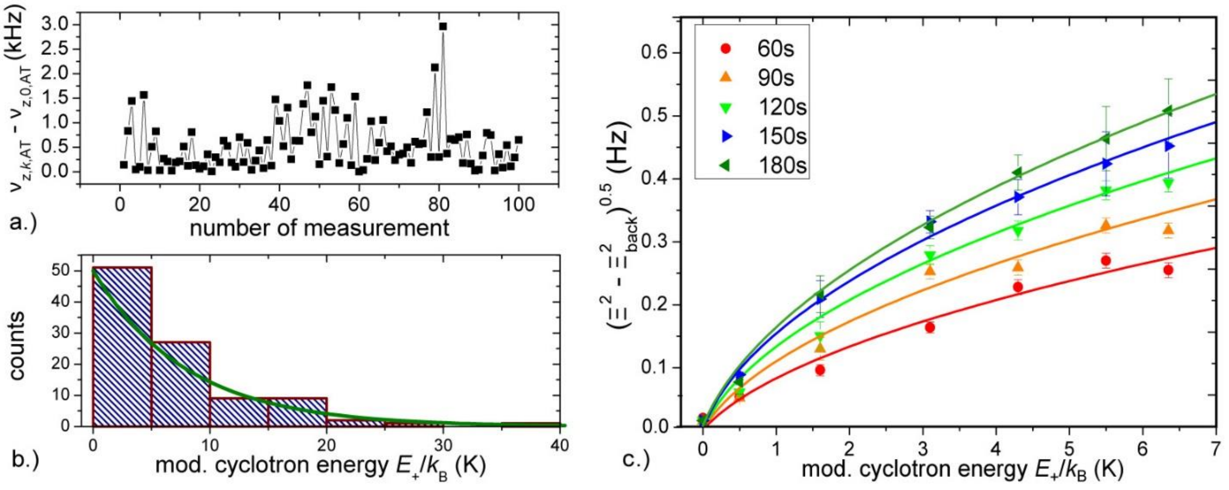

Supplementary Figure 4. Characterization of the modified cyclotron energy. a.) Measured axial frequency shift after thermalization of the cyclotron energy in the co-magnetometer trap. b.) Data shown in a.) as a histogram. From this we extract the temperature of the cyclotron detection system $T_+ = 8.98(74)\text{K}$. c.) Axial frequency fluctuation Ξ_z for different averaging times and different cyclotron energies E_+. From the fits we extract the cyclotron heating rate, as described in the text.

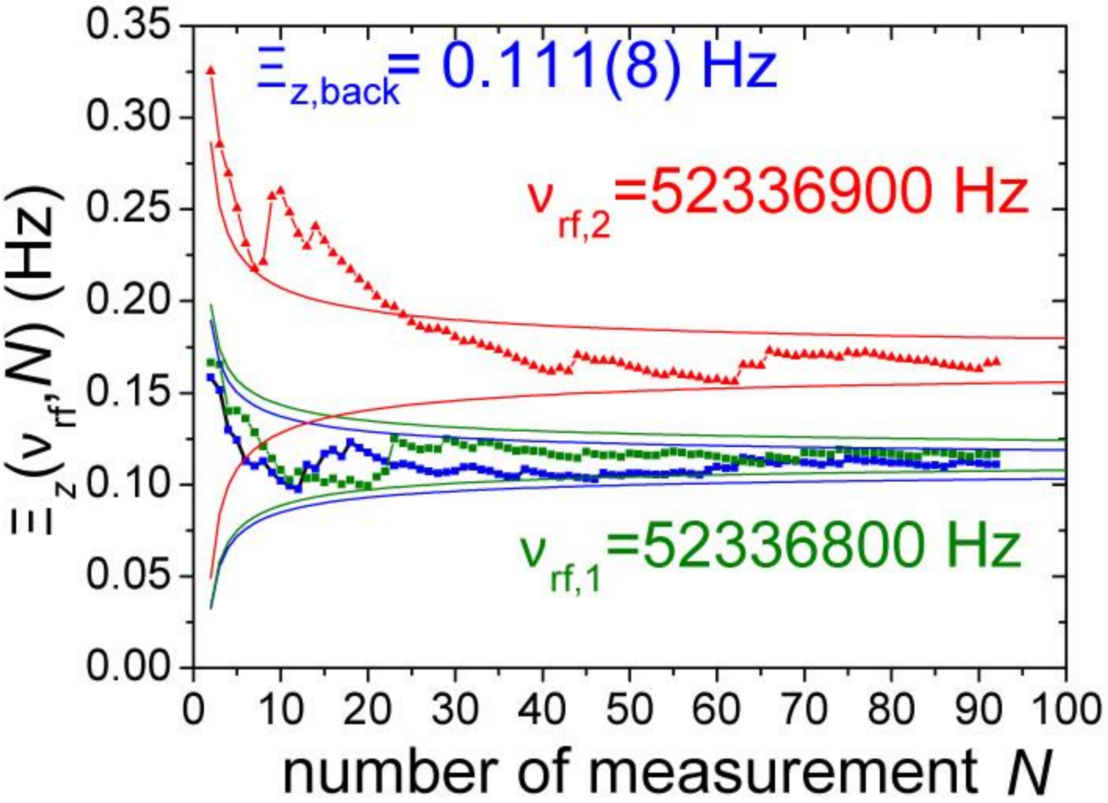

Supplementary Figure 5. Cumulative plot of frequency fluctuations for different spin-flip drive frequencies. The blue data-points represent the background measurement. For the green data points a drive at $\nu_{\mathrm{rf},1} = 52\ 336\ 800$ Hz was irradiated, for the red data points a drive at $\nu_{\mathrm{rf},2} = 52\ 336\ 900$ Hz was applied. The solid lines represent the 68% confidence interval of Ξ_z for the respective measurement.

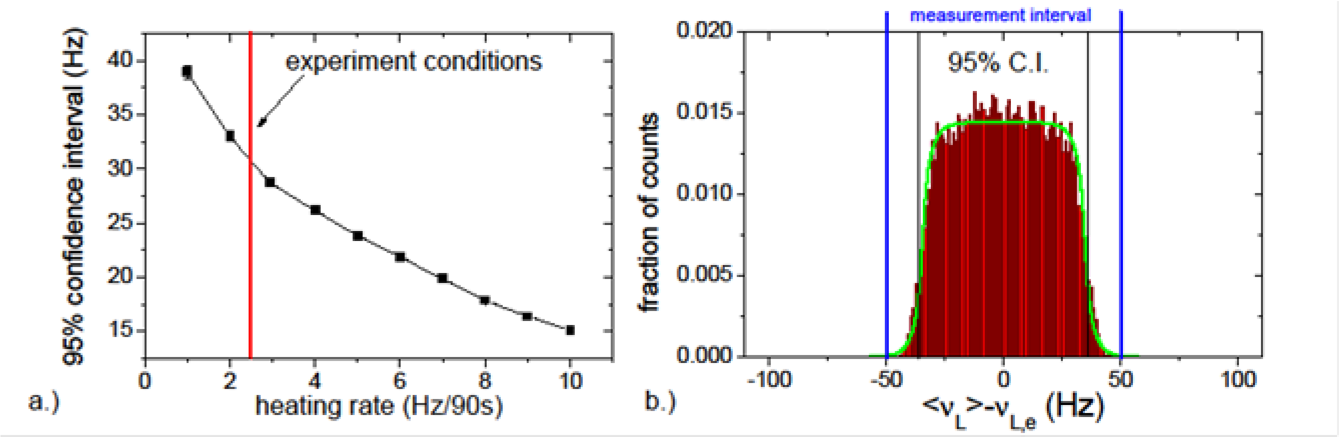

Supplementary Figure 6. Evaluation of the Larmor frequency measurement. a.) 95% confidence level of the distribution $\langle\nu_\mathrm{L}\rangle - \nu_\mathrm{L,e}$ as a function of the magnetron heating rate. The red line indicates the experiment conditions. b.) Distribution of $\langle\nu_\mathrm{L}\rangle - \nu_\mathrm{L,e}$ for the magnetron heating rates which are observed in this experiment. The blue lines indicate the measurement interval, the black lines the 95% confidence interval of $\nu_\mathrm{L,e} = 0.5(\nu_\mathrm{rf,1} + \nu_\mathrm{rf,2})$. The light green line is a result of an explicit calculation, which is consistent with the Monte-Carlo simulated data.

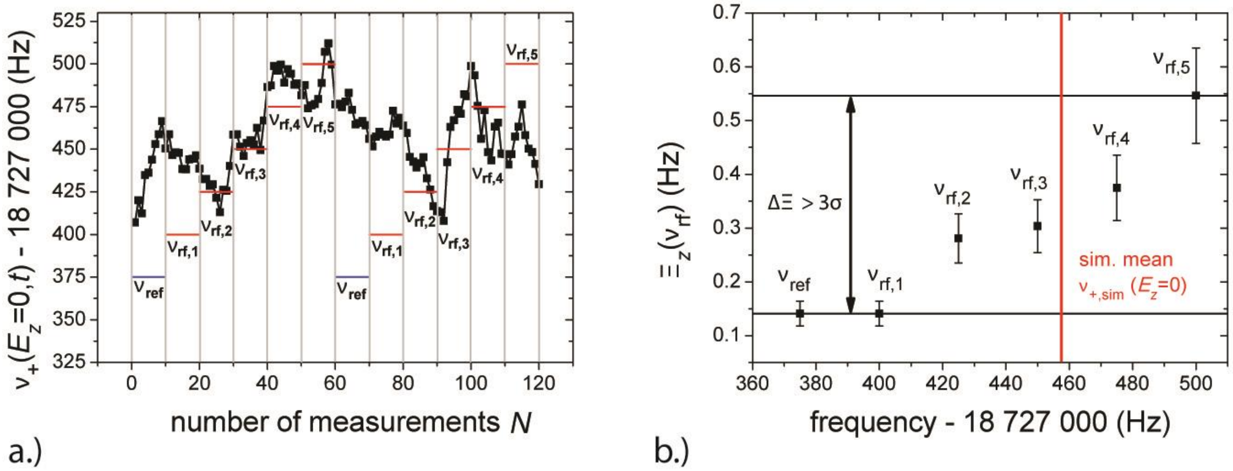

Supplementary Figure 7. Monte-Carlo simulation of the modified cyclotron frequency measurement. a.) Simulated walk of the cyclotron frequency. In this simulation the generating function of the walk has a strength of $\xi_{-} = 10\,\mathrm{Hz}/30\,\mathrm{s}$. b.) Projection of the simulated result to frequency fluctuation $\Xi_z(\nu_{\mathrm{rf}})$. The red line represents the actual mean frequency $\langle \nu_{+,\mathrm{sim}}(E_z = 0, t)\rangle$ of the simulated data.

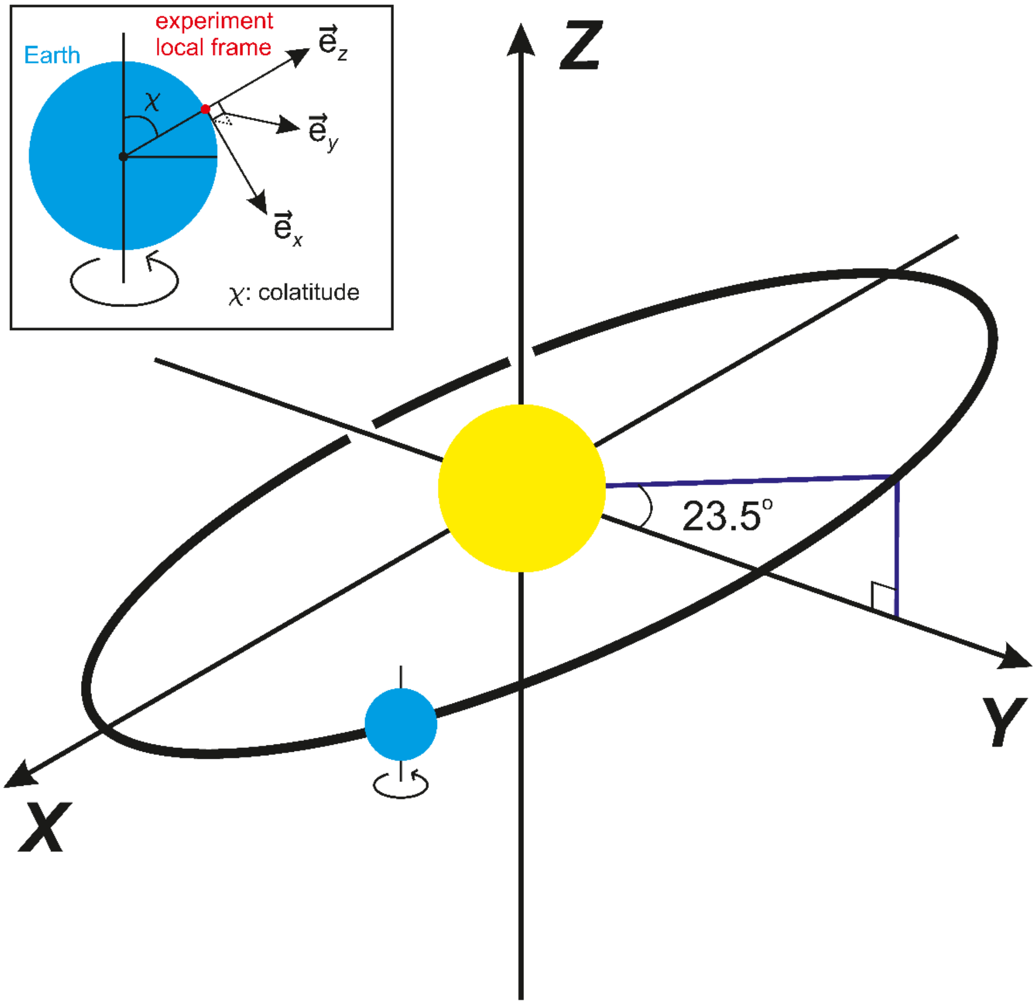

Supplementary Figure 8. Reference frame to define the geometrical conventions of the Standard Model Extension. Capital coefficients represent the SME standard frame which is oriented in parallel to the Earth's rotational axis. The inset on the upper left represents the local experiment frame. For further details, we refer to the text.

Supplementary Discussion

- ## Axial frequency stability

The axial frequency fluctuation $\Xi_{z,\mathrm{back}}$, as described in the main text, is a function of the cyclotron energy E_+ of the trapped antiproton. An Allan deviation plot of the scaling of $\Xi_{z,\mathrm{back}}(\tau_\mathrm{m})$, where τ_m is the averaging time per frequency measurement, is shown in Supplementary Fig. 1.
The black and the red data points represent axial frequency fluctuations measured with a particle at a modified cyclotron energy E_+/k_B of 0.1 K and 1.1 K, respectively. All measurements which are presented in the paper were performed at modified cyclotron energies E_+/k_B below 1.1 K. In measurements of the modified cyclotron frequency we averaged the axial frequency $\nu_{z,\mathrm{AT}}$ for 30 s, as indicated by the blue line, Larmor frequency measurements were carried out at 90 s averaging time represents by the green line.

- ## Line-shapes

The shapes of the modified cyclotron and the Larmor resonance lines in the magnetic bottle are described in detail by L. S. Brown [1]. Our magnetic bottle has a strength of $B_2 = 2.88 \cdot 10^5$ T·m^{-2} at a background magnetic field of $B_0 = 1.227$ T. The effective temperature of our detection system is at $T_z = 8.73(25)$ K. The experiment is operated in the weak coupling range and the line-shape $\chi(\nu_\mathrm{rf},\nu_j,\Delta\nu_j)$ of both, the resonant response lines for modified cyclotron transitions and the Larmor transitions are

$$\chi(\nu_\mathrm{rf},\nu_j,\Delta\nu_j) = \frac{\Theta(\nu_\mathrm{rf}-\nu_j)}{2\pi\Delta\nu_j} \cdot \exp\left(-\frac{\nu_\mathrm{rf}-\nu_j}{\Delta\nu_j}\right), \qquad (1)$$

where $\nu_j$are the resonance frequencies of the modified cyclotron and the Larmor transition, $\nu_{+,\mathrm{cut}}$ and $\nu_{\mathrm{L,cut}}$, respectively, and $\Theta(\nu_\mathrm{rf}-\nu_j)$ is the Heaviside function. The linewidth parameter

$$\Delta\nu_j = \nu_j \frac{B_2}{B_0} \frac{k_\mathrm{B} T_z}{4\pi^2 m_{\bar{\mathrm{p}}} \nu_z^2} \qquad (2)$$

is a measure for the width of the resonance line. Here $k_\mathrm{B}$is the Boltzmann constant, ν_z the axial oscillation frequency and $m_{\bar{\mathrm{p}}}$ the mass of the trapped antiproton.
This line-shape is a direct result of the continuous contact of the particle with the axial detection system. The interaction of the antiproton with the detection system at temperature T_z thermalizes the particle continuously with a correlation time constant of $\tau_\mathrm{c} = 33$ ms. The resulting resonance line is a convolution of unperturbed Lorentz profiles and the Boltzmann distribution $\mathrm{W}(E_z, T_z) = \frac{1}{k_\mathrm{B}T_\mathrm{z}} \cdot \exp(-E_z/(k_\mathrm{B}T_\mathrm{z}))$ [1]. Note that for frequencies $\nu_\mathrm{rf} < \nu_j$ the line-shape function $\chi(\nu_\mathrm{rf}, \nu_j, \Delta\nu_j) = 0$ and for $\varepsilon > 0 \rightarrow \chi(\nu_j + \varepsilon, \nu_j, \Delta\nu_j) = 1/(2\pi\Delta\nu_j)$, which means that for infinitely stable magnetic and electric field conditions the $\frac{d}{d\nu_\mathrm{rf}}\chi(\nu_\mathrm{rf}, \nu_j, \Delta\nu_j)|_{\nu_\mathrm{rf}=\nu_j} \rightarrow \infty$.

- **Line-shape modifications**

The line-shape given above assumes stable magnetic field conditions, so that $\nu_\mathrm{L}(E_z = 0, t)$ is constant for the entire sampling time which is required to resolve the resonance line. However, different drift effects soften the slope of the resonance line and $\frac{d}{d\nu_\mathrm{rf}}\chi(\nu_\mathrm{rf}, \nu_j, \Delta\nu_j)|_{\nu_\mathrm{rf}=\nu_j} = \alpha < \infty$.

These are:

- drifts of the external magnetic field $\xi_\mathrm{B}(t)$,
- voltage drifts of the trap-biasing supplies $\xi_\mathrm{V}(t)$,
- a noise-driven random walk $\xi_+(t)$ in the modified cyclotron mode, and
- a noise-driven random walk $\xi_-(t)$ in the magnetron mode.

By summarizing all these effects to an effective $\xi(t)$, the line-shape modifies to

$$\chi\left(\nu_\mathrm{rf}, \nu_j, \Delta\nu_j, \tau_\mathrm{m}, \xi(t)\right) = \frac{1}{\tau_\mathrm{m}}\int_0^{\tau_m} dt \frac{\Theta(\nu_\mathrm{rf} - (\nu_j + \xi(t)))}{2\pi\Delta\nu_j} \cdot \exp\left(-\frac{\nu_\mathrm{rf} - (\nu_j + \xi(t))}{\Delta\nu_j}\right), \qquad (3)$$

where τ_m is the time required to resolve the cut frequency. Each measured line is consequently a convolution of the unperturbed line over the random processes $\xi_k(t)$. The correlation time constants τ_k of the random drifts $\xi_k(t)$ are large compared to the required measurement time; $\tau_k \gg \tau_\mathrm{m}$. As a result of this discussions of measurement uncertainties need to be based either on Monte-Carlo simulations or distributions approximated by diffusion models.

- **External magnetic field drifts**

Measurements with our co-magnetometer particle, see Supplementary Fig. 2, imply that $\Delta B_0/B_0 < 10^{-8}/\mathrm{h}$ and $\Delta B_0/B_0 < 1.5 \cdot 10^{-8}/(10\ \mathrm{h})$. Intrinsic drifts of the field of the superconducting magnet are small compared to the fractional precision of the measurements quoted here, and are therefore neglected in the following analysis.

- **Voltage stability**

Drifts of the biasing voltages of the trap lead to spatial shifts of the particle in the magnetic bottle and consequently to a shift of the background magnetic field which is experienced by the particle. Voltage drifts on the correction electrodes contribute the most significant shifts in particle position. A voltage drift of 1 mV shifts the particle in the trap by ≈ 1.25 µm. Depending on the equilibrium position z_0 of the particles in the magnetic bottle this leads to position dependent frequency shifts

$$\frac{1}{\nu}\frac{d\nu}{dz}z_0 = 0.49 \cdot \frac{\mathrm{ppm}}{\mu\mathrm{m}} \cdot z_0 \,. \tag{4}$$

To account for this effect we tune the particle carefully to the center of the magnetic bottle. To this end we use the particle and measure the modified cyclotron frequency ν_+, and thus the magnetic field, as a function of position, see Supplementary Fig. 3. The data-set allows us to tune the particle to the bottle center with a spatial resolution of ≈ 1.2 µm.
The voltage stability is directly measured using a reference multimeter and is for a typical measurement time to resolve the Larmor frequency, ≈ 10 h, at 85(4) nV at ≈ 0.75 V. Thus the error in the cyclotron frequency, caused by voltage drifts, is in the worst case of correlated drifts of opposite sign on the two correction electrodes of our 5-electrode compensated Penning trap [2] of order $\Delta\nu_+/\nu_+ < 0.3\ \mathrm{p.p.b.}$.

- **Cyclotron random walk**

The random walk in the cyclotron mode is estimated by measuring the axial frequency as a function of the cyclotron energy E_+. The cyclotron energy is calibrated by thermalizing the particle in the co-magnetometer trap and measuring the axial frequency shift in the analysis trap after each thermalization cycle, see Supplementary Fig. 4 a.) and b.).

$$\nu_z(n_+) = \nu_{z,0} + \frac{h\nu_+}{4\pi^2 m\nu_z}\frac{B_2}{B_0}\left(\left(n_+ + \frac{1}{2}\right)\right). \quad (5)$$

To extract the heating rate $dn_+/dt(E_+)$ we measure the axial frequency fluctuation $\Xi_z(E_+, \tau_\mathrm{m})$ as a function of E_+ and for different averaging times τ_m and fit functions $\Xi_z(E_+, \tau_\mathrm{m}) = \left(\Xi_{\mathrm{z,back}}^2 + \mathrm{d}n_+/\mathrm{dt}\,(E_+) \cdot \Delta\nu_{z,+}^2 \cdot \tau_\mathrm{m}/\sqrt{2}\right)^{0.5}$ to the measured data, see Supplementary Fig. 4 c.). For different modified cyclotron energies, we extract a heating rate of $\mathrm{d}n_+/\mathrm{dt}\,(E_+) = 0.08 \cdot E_+/k_\mathrm{B}(\mathrm{s}\cdot\mathrm{K})^{-1}$. All measurements reported here were carried out with particles at cyclotron energies $E_+/k_\mathrm{B} < 1.1$ K. One cyclotron quantum transition changes the magnetic field experienced by the particle by $\Delta B_0/B_0 = 3 \cdot 10^{-10}$, and for typical measurement times of order 1 h to 20 h the cyclotron walk contributes at maximum a fractional shift in the cyclotron frequency of 20 p.p.b.. Within the experimental resolution reported here the effect of the random walk $\xi_+(t)$ in the cyclotron mode is negligibly small.

- ## Magnetron random walk

To characterize the magnetron heating rate dn_-/dt during Larmor frequency measurements we measure first the cyclotron frequency $\nu_{+,1}$, perform the scan of the Larmor frequency ν_L and measure the cyclotron frequency again $\nu_{+,2}$. Since the magnetic field is stable at the level $\Delta B_0/B_0 < 1.5 \cdot 10^{-8}/(10\ \mathrm{h})$, and a typical measurement to resolve the Larmor frequency takes 10 h, the measured cyclotron frequency difference can be accounted solely to a drift in the magnetron mode. We obtain from a set of 20 long-term measurements a distribution

$$\frac{dn_-}{dt} = \sqrt{2}\frac{(\nu_{+,2} - \nu_{+,1})^2}{\tau_\mathrm{m}(\Delta\nu_+/\Delta n_-)^2}, \quad (6)$$

with $\Delta\nu_+/\Delta n_- = 0.0024$ Hz being the cyclotron frequency shift per magnetron quantum transition. From the width of the distribution we obtain $dn_-/dt = 18.5(3.0) \cdot 10^3/\mathrm{s}$. Thus, the magnetron random walk $\xi_-(t)$ in the magnetron mode leads to an average root mean square drift of the cyclotron frequency at $\Delta\nu_+/\nu_+(t) = 8.4(6) \cdot 10^{-7}\ \sqrt{t}/\sqrt{h}$, and constitutes the dominant drift mechanism which softens the slope of the resonance. During our g-factor measurements, we are able to constrain the random-walk based on the observed frequency fluctuations and the differences of the measured cyclotron frequencies. The remaining uncertainty in the evolution of the magnetron radius is reflected in the uncertainty of the measured Larmor and cyclotron frequencies obtained using the analytical and Monte-Carlo evaluation methods decribed below.

- **Measurement of the spin-resonance**

To detect spin transitions we use the statistical detection technique which is described in [3]. We

1. measure the axial frequency $\nu_{z,4k-4}$,
2. irradiate a reference drive with $\nu_{\mathrm{rf}} < \nu_{\mathrm{L}}$,
3. measure the axial frequency $\nu_{z,4k-3}$,
4. irradiate a resonant drive close to resonance $\nu_{\mathrm{rf},1} \approx \nu_{\mathrm{L}}(E_z = 0)$,
5. measure the axial frequency $\nu_{z,4k-2}$,
6. irradiate another drive close to resonance $\nu_{\mathrm{rf},2} \approx \nu_{\mathrm{L}}(E_z = 0)$,
7. and conclude the sequence by another measurement of the axial frequency $\nu_{z,4k-1}$.

This sequence is repeated for $N > 80$ times and subsequently we evaluate:

1. the standard deviation $\sigma(\nu_{z,4k-4} - \nu_{z,4k-3}) \coloneqq \Xi_{z,\mathrm{back}}$ for the off-resonant reference drive,
2. the standard deviation $\sigma(\nu_{z,4k-3} - \nu_{z,4k-2}) \coloneqq \Xi_z(\nu_{\mathrm{rf},1})$ for the first drive close to $\nu_{\mathrm{L}}(E_z = 0)$ as well as
3. the standard deviation $\sigma(\nu_{z,4k-2} - \nu_{z,4k-1}) \coloneqq \Xi_z(\nu_{\mathrm{rf},2})$ for the second drive close to $\nu_{\mathrm{L}}(E_z = 0)$.

In the first case we obtain the reference fluctuation $\Xi_{z,\mathrm{back}}$. In the second and the third case the potentially induced spin transitions add frequency jumps $\Delta\nu_{z,\mathrm{SF}} = 183$ mHz to the background fluctuation and the measured axial frequency fluctuations $\Xi_z(\nu_{\mathrm{rf},1})$ and $\Xi_z(\nu_{\mathrm{rf},2})$ become

$$\Xi_z(\nu_{\mathrm{rf},k}) = \sqrt{\Xi_{\mathrm{z,back}}^2 + P_{\mathrm{SF}}(\nu_{\mathrm{rf},k}, \nu_j, \Delta\nu_j)\Delta\nu_{z,\mathrm{SF}}^2}\,, \tag{7}$$

where $P_{\mathrm{SF}}(\nu_{\mathrm{rf},k}, \nu_j, \Delta\nu_j)$ is the frequency dependent spin flip probability [1] at a given drive strength Ω_{R} of a radio frequency drive at frequency ν_{rf} which is irradiated for a time $t_0 = 10$ s:

$$P_{\mathrm{SF}}(\nu_{\mathrm{rf},k}, \nu_j, \Delta\nu_j) = \frac{1}{2}\left(1 - \exp\left(-\frac{1}{2}\Omega_{\mathrm{R}}^2 t_0 \chi(\nu_{\mathrm{rf},k}, \nu_j, \Delta\nu_j)\right)\right). \tag{8}$$

Supplementary Fig. 5 shows the cumulative background fluctuation $\Xi_{z,\text{back}}$ (blue data points) as well as $\Xi_z(\nu_{\text{rf},1} = 52\,336\,800\text{ Hz})$ (green data points) and $\Xi_z(\nu_{\text{rf},2} = 52\,336\,900\text{ Hz})$ (red data points) as a function of measurement number N, the solid lines describe the calculated error bands $\Delta\sigma(\nu_{\text{rf},k}, N) = \Xi_z(\nu_{\text{rf},k})/(2N-2)^{0.5}$ of the respective measurement. The spinflip drive excites transitions at $\Xi_z(\nu_{\text{rf},2} = 52\,336\,900\text{ Hz})$ while the points at $\Xi_z(\nu_{\text{rf},1} = 52\,336\,800\text{ Hz})$ are consistent with the background measurement. In this measurement we accumulated 93 data points at a background fluctuation of $\Xi_{z,\text{back}} = 0.111(8)$ Hz, while $\Xi_z(\nu_{\text{rf},1}) = 0.116(8)$ Hz and $\Xi_z(\nu_{\text{rf},2}) = 0.168(13)$ Hz. The statistical significance of $\Xi_z(\nu_{\text{rf},2}) - \Xi_z(\nu_{\text{rf},1})$ is at $> 3.5\,\sigma$. We extract as Larmor frequency $\nu_{\text{L}} = 0.5 \cdot (\nu_{\text{rf},1} + \nu_{\text{rf},2}) = 52\,336\,850$ Hz, the discussion of the uncertainty which we quote to this value is given below.

- **Undersampling**

In some measurements, as e.g. the one shown in Supplementary Fig. 5 of the spin resonance the cut frequency was undersampled, which means that the diffusion of $\nu_+(E_z = 0, \tau_{\text{m}}) - \nu_+(E_z = 0,0)$ was smaller than the sampling interval $\Delta\nu_{\text{rf}}$ which was chosen in the frequency scan.

Under these conditions we measure within the 68% confidence level $\Xi_z(\nu_{\text{rf},1}) = \Xi_{z,\text{back}}$ and $(\Xi_z(\nu_{\text{rf},2}) - \Xi_{z,\text{back}})/\sigma(\Xi_z(\nu_{\text{rf},2}), \Xi_{z,\text{back}}) > 3.5$. From the experiment sequence we have measured parameters $\nu_{+,1}$, the information from the Larmor measurement, as well as $\nu_{+,2}$. To give in this case of undersampling an appropriate error estimate, the following case needs to be discussed: Given the input parameters of the experiment $\nu_{+,1}$ and $\nu_{+,2}$, including their uncertainties, an appropriate estimate of the uncertainty needs to be derived.
We run random walk Monte-Carlo simulations with defined parameters τ_{m} and $\nu_{+,1} - \nu_{+,2}$.
The start frequency $\nu_{+,1}$ and the heating rate $\xi_-(t)$ are varied. We accept random walks which reproduce our result that

- within the 68% C.L. $\Xi_z(\nu_{\text{rf},1}) = \Xi_{z,\text{back}}$ and
- $(\Xi_z(\nu_{\text{rf},2}) - \Xi_{z,\text{back}})/\sigma(\Xi_z(\nu_{\text{rf},2}), \Xi_{z,\text{back}}) > 3.5$.

We calculate the mean frequency of the simulated walk $\langle \nu_{\mathrm{L}} \rangle$ and compare to the frequency $\nu_{\mathrm{L},e} \equiv 0.5(\nu_{\mathrm{rf},1} + \nu_{\mathrm{rf},2})$ which would have been extracted from the measurement. Based on 1000 simulations for each parameter $\nu_{+,1}$ we evaluate the distribution $\langle \nu_{\mathrm{L}} \rangle - \nu_{\mathrm{L,e}}$, integrate $\nu_{+,1}$ in boundaries $\nu_{\mathrm{rf},1}$ to $\nu_{\mathrm{rf},2}$ and calculate the 95% confidence interval of the integrated distribution. Supplementary Fig. 6 a.) shows the scaling of the 95% confidence level of the distributions $\langle \nu_{\mathrm{L}} \rangle - \nu_{\mathrm{L,e}}$ for different magnetron heating rates dn_{-}/dt. The red line indicates the experimental conditions. Note that the uncertainty of the distribution $\langle \nu_{\mathrm{L}} \rangle - \nu_{\mathrm{L,e}}$ increases with reduced heating rate, which is caused by the boundary conditions $\nu_{+}(t = 0) = \nu_{+,1}$ and $\nu_{+}(t = \tau_{\mathrm{m}}) = \nu_{+,2}$. Walks which fulfil the boundary conditions at high heating rates trace the frequency interval $\nu_{\mathrm{rf},2} - \nu_{\mathrm{rf},1}$ more equally than walks at low heating rate. The probability to reproduce in case of a strong walk the true mean frequency of the walk by the arithmetic mean is thus enhanced. Supplementary Fig. 6 b.) displays the integrated distribution of $\langle \nu_{\mathrm{L}} \rangle - \nu_{\mathrm{L,e}}$ for the magnetron heating rates $dn_{-}/dt = 18.5(3.0) \cdot 10^{3}\ \mathrm{s}^{-1}$, which are observed in this experiment. The blue lines indicate the measurement interval, the black lines the 95% confidence level of $\nu_{\mathrm{L,e}} = 0.5(\nu_{\mathrm{rf},1} + \nu_{\mathrm{rf},2})$. Based on this evaluation we define the 95% confidence interval as $\Delta\nu_{\mathrm{L}} = 33$ Hz. A purely analytical treatment to derive the distribution $w(\langle \nu_{\mathrm{L}} \rangle - \nu_{\mathrm{L,e}})$ shown in Supplementary Fig. 6 b.) requires integration of the mean of distributions in the resolved frequency interval, derivation of a scaling function which reflects the fraction of walks which meet the boundary conditions of the experiment for different heating rates, the evaluation of $\langle \nu_{\mathrm{L}} \rangle - \nu_{\mathrm{L,e}}$ based on a diffusion model and the integration of the results over all possible heating rates. Rather than explicitly quoting the formulas we show the result of such an analytical treatment together with the Monte-Carlo simulated data, see light green line in Supplementary Fig. 6 b.).

• Measurement of the modified cyclotron frequency

Compared to the spin system with eigenstates $\pm\hbar/2$ the quantum numbers of the cyclotron oscillator are $n_{+} \geq 1$. Many transitions can be induced within one excitation cycle, and thus, the resolution of the slope around $E_z = 0$ affords significantly shorter measurement time. The measurement procedure to determine the cyclotron frequency is described in the main text. We measure the axial frequency fluctuation $\Xi_z(\nu_{\mathrm{rf}})$ while irradiating a radial rf-drive at $\nu_{\mathrm{rf},k}$. One analysis method was described in the main text, another one is based on Monte-Carlo simulations. Based on the results $\Xi_z(\nu_{\mathrm{rf}})$ we reconstruct, using Monte-Carlo simulations, the distribution w of cyclotron frequencies during the measurement sequence, evaluate the distribution of mean values $\widetilde{w}$ which results from w, calculate the expectation value of $\widetilde{w}$ and quote its 95% confidence level as the uncertainty.
To visualize the Monte-Carlo based reconstruction of w, Supplementary Fig. 7 illustrates a basic example.

The black squares in Supplementary Fig. 7 a.) show results of a simulated walk of the cyclotron cut frequency, the red lines indicate the excitation frequencies $\nu_{\mathrm{rf},k}$, and the blue lines indicate the off-resonance frequency ν_{ref}. In Supplementary Fig. 7 b.) the projection to measured frequency fluctuation is shown. We perform simulations for different start/stop frequencies and different heating rates of the walks and select walks which reproduce the measured data points within their 68% error bars. For each heating rate we obtain a sub-distribution $\widetilde{w}_k$. We integrate over all possible sub-distributions $\widetilde{w}_k$ to obtain a final distribution of cyclotron mean values, calculate the expectation value of $\widetilde{w}$ and quote its 95% confidence level as the uncertainty.

- **Summary of measured frequencies**

The table below summarizes all measured modified cyclotron and Larmor frequencies which enter the g-factor evaluation. For all g-factor measurements, the axial frequency was at 674 832 (9) Hz.

g-factor	$\nu_{+,1}$ (Hz)	ν_{L} (Hz)	$\nu_{+,2}$ (Hz)
1	18 727 430 (30)	52 336 760 (155)	18 727 454 (71)
2	18 727 452 (35)	52 336 800 (166)	18 727 338 (14)
3	18 727 438 (11)	52 336 850 (33)	18 727 467 (32)
4	18 727 476 (34)	52 336 900 (77)	18 727 513 (11)
5	18 727 452 (33)	52 336 895 (86)	18 727 400 (33)
6	18 727 601 (48)	52 337 350 (77)	18 727 664 (48)

- **Standard Model Extension (SME)**

In this section, the sensitivity of our experiment with respect to the parameters of the Standard Model Extension (SME) [4,5,6], a model which allows discussion of the sensitivity of experiments with respect to CPT violating coefficients derived based on an effective field theory, is presented. In this evaluation we apply the formalism of a very recent comprehensive publication on the application of the SME to Penning trap based magnetic moment measurements [6]. Details to obtain constraints on SME coefficients by comparing results of experiments performed at different magnetic field strengths, orientations, and locations are outlined in Eq. (65), Eq. (67), Eq. (76) and Eq. (80) of [6].

To derive new constraints on the coefficients $\tilde{b}_{\mathrm{p}}^{Z}$, $\tilde{b}_{\mathrm{F,P}}^{XX}+\tilde{b}_{\mathrm{F,P}}^{YY}$, and $\tilde{b}_{\mathrm{F,P}}^{ZZ}$ for protons, and $\tilde{b}_{\mathrm{p}}^{*Z}$, $\tilde{b}_{\mathrm{F,P}}^{*XX}+\tilde{b}_{\mathrm{F,P}}^{*YY}$, and $\tilde{b}_{\mathrm{F,P}}^{*ZZ}$ for antiprotons, we follow [6] and compare our 2014 $(g/2)_{\mathrm{p}}=2.792847350(9)$ g-factor measurement, which was performed at Mainz, to the antiproton g-factor measurement which

is presented here. Both experiments are in horizontal design. As local coordinate systems to define the orientations of our magnets we use the local zenith as $\vec{e}_z = \cos(\varphi)\sin(\chi)\,\vec{e}_X + \sin(\varphi)\sin(\chi)\,\vec{e}_Y + \cos(\chi)\,\vec{e}_Z$, here small indices represent the local laboratory frame and capital indices represent the standard frame used in the SME, with *Z*-component along the Earth's rotational axis (compare Supplementary Fig. 8). The angle χ is the local colatitude, $\chi_\mathrm{C} \approx 44°$ for the CERN experiment and $\chi_\mathrm{M} \approx 40°$ for the Mainz experiment. As local *y*-component we use the vector which points eastwards, as local *x*-component we define the vector which points southwards. We assume that the azimuthal component φ averages out due to the Earth's rotation. In the local Mainz experiment frame the magnetic field is oriented southwards $\gamma_\mathrm{M} \approx 0°$, in the CERN experiment the axis of the magnet is oriented $\gamma_\mathrm{C} \approx 120°$ with respect to the local *x*-axis, where we define counter-clock-wise rotation as positive.

To derive constraints on SME coefficients based on our two measurement, the *g*-factors of the proton $(g/2)_\mathrm{p}$ and the antiproton $(g/2)_{\bar{\mathrm{p}}}$ can be compared as

$$\left(\frac{g}{2}\right)_\mathrm{p} - \left(\frac{g}{2}\right)_{\bar{\mathrm{p}}} = \frac{2}{\omega_\mathrm{c}^\mathrm{p}\omega_\mathrm{c}^{\bar{\mathrm{p}}}}\left(\Sigma\omega_\mathrm{c}^\mathrm{p}\Delta\omega_\mathrm{a}^\mathrm{p} - \Delta\omega_\mathrm{c}^\mathrm{p}\Sigma\omega_\mathrm{a}^\mathrm{p}\right), \tag{9}$$

where,

$$\Delta\omega_\mathrm{c}^\mathrm{p} = \frac{1}{2}\left(\omega_\mathrm{c}^\mathrm{p} - \omega_\mathrm{c}^{\bar{\mathrm{p}}}\right) \tag{10}$$

$$\Sigma\omega_\mathrm{c}^\mathrm{p} = \frac{1}{2}\left(\omega_\mathrm{c}^\mathrm{p} + \omega_\mathrm{c}^{\bar{\mathrm{p}}}\right) \tag{11}$$

$$\Delta\omega_\mathrm{a}^\mathrm{p} = \frac{1}{2}\left(\delta\omega_\mathrm{a}^\mathrm{p} - \delta\omega_\mathrm{a}^{\bar{\mathrm{p}}}\right) \tag{12}$$

$$\Sigma\omega_\mathrm{a}^\mathrm{p} = \frac{1}{2}\left(\delta\omega_\mathrm{a}^\mathrm{p} + \delta\omega_\mathrm{a}^{\bar{\mathrm{p}}}\right). \tag{13}$$

$\omega_\mathrm{c}^\mathrm{p}$ and $\omega_\mathrm{c}^{\bar{\mathrm{p}}}$ are the cyclotron frequencies of the proton and the antiproton, respectively, while $\delta\omega_\mathrm{a}^\mathrm{p}$ and $\delta\omega_\mathrm{a}^{\bar{\mathrm{p}}}$ are the shifts in the anomaly frequencies due to hypothetical CPT violating effects. For explicit expressions of $\Delta\omega_\mathrm{a}^\mathrm{p}$ and $\Sigma\omega_\mathrm{a}^\mathrm{p}$ we refer to Eq. (68) and Eq. (69) in [6]. This gives:

$$\begin{aligned}\Sigma\omega_\mathrm{a}^\mathrm{p} = &-\tilde{b}_\mathrm{p}^{Z}\sin(\chi_\mathrm{M}) - \frac{1}{2}\left(\tilde{b}_\mathrm{F,P}^{XX} + \tilde{b}_\mathrm{F,P}^{YY}\right)B\cos^2(\chi_\mathrm{M}) - \tilde{b}_\mathrm{F,P}^{ZZ}B\sin^2(\chi_\mathrm{M})\\ &+\tilde{b}_\mathrm{p}^{*Z}\sin(\chi_\mathrm{C})\cos(\gamma_\mathrm{C}) + \frac{1}{2}\left(\tilde{b}_\mathrm{F,P}^{*XX} + \tilde{b}_\mathrm{F,P}^{*YY}\right)B^*\left(\cos^2(\chi_\mathrm{C})\cos^2(\gamma_\mathrm{C}) + \sin^2(\gamma_\mathrm{C})\right)\\ &+\tilde{b}_\mathrm{F,P}^{*ZZ}B^*\sin^2(\chi_\mathrm{C})\cos^2(\gamma_\mathrm{C})\end{aligned} \tag{14}$$

and

$$\Delta\omega_{\mathrm{a}}^{\mathrm{p}} = -\tilde{b}_{\mathrm{p}}^{Z}\sin(\chi_{\mathrm{M}}) - \frac{1}{2}\left(\tilde{b}_{\mathrm{F,P}}^{XX} + \tilde{b}_{\mathrm{F,P}}^{YY}\right)B\cos^{2}(\chi_{\mathrm{M}}) - \tilde{b}_{\mathrm{F,P}}^{ZZ}B\sin^{2}(\chi_{\mathrm{M}})$$

$$-\tilde{b}_{\mathrm{p}}^{*Z}\sin(\chi_{\mathrm{C}})\cos(\gamma_{\mathrm{C}}) - \frac{1}{2}\left(\tilde{b}_{\mathrm{F,P}}^{*XX} + \tilde{b}_{\mathrm{F,P}}^{*YY}\right)B^{*}\left(\cos^{2}(\chi_{\mathrm{C}})\cos^{2}(\gamma_{\mathrm{C}}) + \sin^{2}(\gamma_{\mathrm{C}})\right)$$

$$-\tilde{b}_{\mathrm{F,P}}^{*ZZ}B^{*}\sin^{2}(\chi_{\mathrm{C}})\cos^{2}(\gamma_{\mathrm{C}}) \qquad (15)$$

By inserting these equations together with Eq. (10) and Eq. (11) into Eq. (9), we derive the following SME coefficients:

SME coefficient	[6]	this work
$\lvert\tilde{b}_{\mathrm{p}}^{Z}\rvert$	$< 2 \cdot 10^{-21}$ GeV	$< 2.1 \cdot 10^{-22}$ GeV
$\lvert\tilde{b}_{\mathrm{p}}^{*Z}\rvert$	$< 6 \cdot 10^{-21}$ GeV	$< 2.5 \cdot 10^{-22}$ GeV
$\lvert\tilde{b}_{\mathrm{F,P}}^{XX} + \tilde{b}_{\mathrm{F,P}}^{YY}\rvert$	$< 1 \cdot 10^{-5}$ GeV^{-1}	$< 1.2 \cdot 10^{-6}$ GeV^{-1}
$\lvert\tilde{b}_{\mathrm{F,P}}^{ZZ}\rvert$	$< 1 \cdot 10^{-5}$ GeV^{-1}	$< 8.8 \cdot 10^{-7}$ GeV^{-1}
$\lvert\tilde{b}_{\mathrm{F,P}}^{*XX} + \tilde{b}_{\mathrm{F,P}}^{*YY}\rvert$	$< 2 \cdot 10^{-5}$ GeV^{-1}	$< 8.3 \cdot 10^{-7}$ GeV^{-1}
$\lvert\tilde{b}_{\mathrm{F,P}}^{*ZZ}\rvert$	$< 8 \cdot 10^{-6}$ GeV^{-1}	$< 3.0 \cdot 10^{-6}$ GeV^{-1}

For comparison we also list the coefficients published in [6]. The leading coefficients are improved by a factor of 11 and 22, respectively. Note that the g-factor data on which we base this evaluation are at 95% confidence level, which was considered in the data evaluation. In [6] it is assumed that the experimental uncertainties are at 68% confidence level and to approximate the 95% confidence level the coefficients were constrained using twice the quoted uncertainty.

Supplementary References:

[1] Brown, L. S., Geonium lineshape. *Ann. Phys*. **159**, 62-98 (1985).
[2] Gabrielse, G., Haarsma, L., and Rolston, L. S., Open-endcap Penning traps for high precision experiments. *Int. J. Mass Spectrom*. **88**, 319-332 (1989).
[3] Ulmer, S. et al., Observation of spin flips with a single trapped proton. *Phys. Rev. Lett*. **106**, 253001 (2011).
[4] Kostelecky, V. A., and Russell N. Data-tables on Lorentz and CPT Violation, *Rev. Mod. Phys*. **83**, 11-31 (2011).

[5] Bluhm, R. et al., CPT and Lorentz tests in Penning traps. *Phys. Rev. D.* **57**, 3932-3943 (1998).
[6] Ding, Y., Kostelecký, V. A., Lorentz-violating spinor electrodynamics and Penning traps. *Phys. Rev. D.* **94**, 056008 (2016).
